# Engineering approaches to manipulate osteoclast behavior for bone regeneration

**DOI:** 10.1016/j.mtbio.2024.101043

**Published:** 2024-04-03

**Authors:** Xin Cheng, Wenzhi Tian, Jianhua Yang, Jiamian Wang, Yang Zhang

**Affiliations:** a Department of Stomatology, Shenzhen University General Hospital, Shenzhen University Clinical Medical Academy, 1098 Xueyuan Road, Shenzhen 518055, Guangdong Province, China; b Jilin University, Jilin Province Key Lab Tooth Dev & Bone Remodeling, School and Hospital of Stomatology, Department of Oral Pathology, Changchun 130041, Jilin Province, China; c Longgang District People's Hospital of Shenzhen & the Second Affiliated Hospital, The Chinese University of Hong Kong, Shenzhen 518172, Guangdong province, China; d National Innovation Center for Advanced Medical Devices, Shenzhen 518000, Guangdong Province, China; e School of Dentistry, Shenzhen University Medical School, 1088 Xueyuan Road, Shenzhen 518055, Guangdong Province, China; f School of Biomedical Engineering, Shenzhen University Medical School, 1088 Xueyuan Road, Shenzhen 518055, Guangdong Province, China

**Keywords:** Osteoclasts, Drug delivery, Biomaterials, Bone coupling, Bone regeneration

## Abstract

Extensive research has delved into the multifaceted roles of osteoclasts beyond their traditional function in bone resorption in recent years, uncovering their significant influence on bone formation. This shift in understanding has spurred investigations into engineering strategies aimed at leveraging osteoclasts to not only inhibit bone resorption but also facilitate bone regeneration. This review seeks to comprehensively examine the mechanisms by which osteoclasts impact bone metabolism. Additionally, it explores various engineering methodologies, including the modification of bioactive material properties, localized drug delivery, and the introduction of exogenous cells, assessing their potential and mechanisms in aiding bone repair by targeting osteoclasts. Finally, the review proposes current limitations and future routes for manipulating osteoclasts through biological and material cues to facilitate bone repair.

## Graphical abstract

Key strategies to manipulate osteoclast behavior for bone regeneration. The osteoclast behavior can be modulated by biomaterial properties, mechanical stimuli, natural products and synthetic drugs, and cells and their derivates such as exosomes and growth factors. Bone regeneration capacity can be further regulated by the altered activities of osteoclasts through the bone remodeling process.

**Figure:**
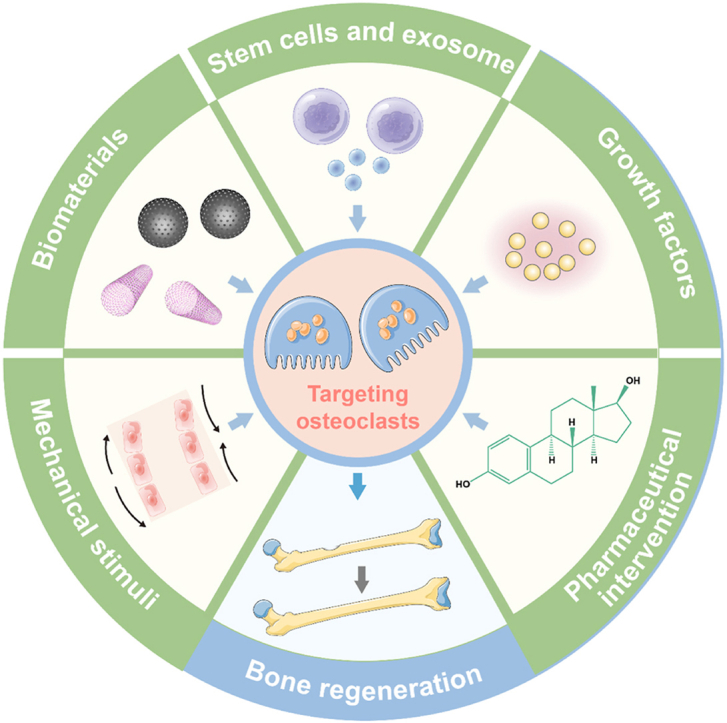

## 1 Introduction

Bone tissue undergoes continuous remodeling to withstand different loads and store essential minerals like phosphate and calcium (Ca^2+^). This remodeling process involves osteoclasts removing damaged bone and osteoblasts forming new bone [1]. Maintaining bone homeostasis relies on the delicate equilibrium between bone resorption and bone formation. However, certain bone diseases, including periodontal disease, rheumatoid arthritis, osteoporosis, and myeloma, disrupt this balance by causing excessive osteoclastic bone resorption. Consequently, bone remodeling becomes impaired, resulting in weakened bone structure and an increased susceptibility to fractures [2].

**Fig. 1:**
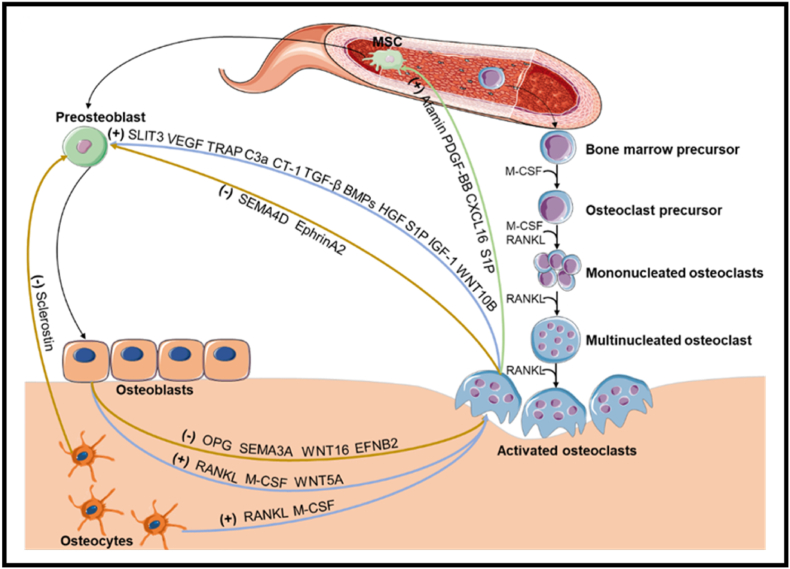
A scheme of osteoclast differentiation and the interaction between osteoclasts and other bone resident cells in the bone microenvironment. Mature (activated) osteoclasts develop from the fusion of multiple individual peripheral blood-borne mononuclear cells, and are characterized by cytomembrane, multiple nuclei and ruffled border responsible for the degradation of bone matrix. Osteoclasts, pre-osteoblasts, osteoblasts, and osteocytes have reciprocal interactions by different cytokines to maintain bone homeostasis. Plus signs and minus signs indicate positive and negative regulators, respectively.

The interaction between osteoclasts and osteoblasts, known as bone coupling, plays a crucial role in maintaining bone homeostasis [3]. In this process, osteoblasts release various soluble factors that can have different effects on osteoclast behavior (Fig. 1). Semaphoring 3A (Sema3A), Wnt family member 16 (Wnt16), and ephrin B2 expressed by osteoblasts have been found to inhibit osteoclast differentiation, while Wnt5a has a positive effect [4]. On the other hand, osteoclast-derived sphingosine 1 phosphate (S1P) and Wnt10b have been reported to stimulate osteogenesis, while semaphoring 4D (Sema4D) suppresses osteoblastic differentiation [[5], [6], [7]]. Furthermore, osteoclasts also indirectly affect bone formation by playing a role in angiogenesis. Pre-osteoclasts have been shown to induce blood vessel formation by secreting platelet-derived growth factor BB (PDGF-BB) during the bone remodeling process [8]. Additionally, matrix metalloproteinase-9 (MMP-9) is not only important for osteoclast resorption and invasion but also contributes to osteoclast-mediated angiogenesis in skeletal homeostasis [9]. As a result, osteoclasts have emerged as a pivotal focus in diverse clinical therapies designed to mitigate bone loss and fractures. Within these investigations, the modulation of osteoclast behavior is meticulously orchestrated in varying spatial and temporal patterns, strategically tailored to induce desired catabolic and anabolic effects under different physiological conditions.

Various types of biomaterials with different physical and chemical properties, including polymers, ceramics, and composite biomaterials, have been developed to aid in bone regeneration [[10], [11], [12]]. However, these approaches predominantly focus on promoting osteoblastic differentiation, and there is limited research on biomaterials or drug delivery systems that specifically target osteoclast development to enhance bone formation. Clinically, anti-resorptive agents like bisphosphonates and denosumab have been widely used to target osteoclasts and manage conditions such as osteoporosis and other bone disorders to prevent bone loss. However, traditional delivery methods (oral and parenteral routes) are associated with several undesirable side effects. For example, bisphosphonates can lead to complications like mandibular osteonecrosis and renal dysfunction [13], while denosumab can cause endocarditis and pancreatitis [14]. Moreover, oral administration is often ineffective as drugs tend to be hydrolyzed in the gastrointestinal tract, resulting in reduced bioavailability at the desired sites. Parenteral administration via intravenous delivery has also been linked to influenza-like myalgic symptoms in bisphosphonate-treated postmenopausal women with osteoporosis [15]. Consequently, advanced engineering approaches using biomaterials to achieve controlled drug release and manipulate osteoclast behavior has gained prominence in recent decades. In the subsequent sections of this review, we provide a summary of osteoclast biology in bone remodeling, discuss engineering approaches developed for targeting osteoclasts to aid in bone repair, and present an overview of current limitations and prospects.

## 2 The biology and role of osteoclasts in bone development

### 2.1 Osteoclasts in bone remodeling

As a mineralized connective tissue, bone is comprised of four types of cells: osteoblasts, bone lining cells, osteocytes, and osteoclasts. Bone remolding is necessary to maintain the structural integrity of bone and subserve its metabolic functions under physiological conditions. A regular bone remodeling cycle generally starts with microfracture and mechanical loading in the bone microenvironment, the bone fatigue can be detected by osteocytes, which are derived from osteoblasts and embedded in the bone matrix, osteocytes then produce and release cytokines such as receptor activator of nuclear factor kappa-B (NF-κB) ligand (RANKL) and macrophage colony-stimulating factor (M-CSF) to recruit osteoclast precursors and promote their differentiation into multinucleated mature osteoclasts [16]. Although bone lining cells are derived from osteoblasts and located on the bone surfaces where neither bone formation nor resorption proceeds [17], gap junctions can be observed between them and osteocytes [18], and there is evidence that they can intercept the direct interaction between osteoclasts and bone matrix to prevent bone resorption [19]. Bone matrix can not only provide mechanical support to bone cells because of its complex and organized framework, but also participate in bone remodeling via releasing several molecules [20]. The αvβ3 and α2β1 integrins expressed by osteoclasts can bind to bone matrix proteins (such as osteopontin and bone sialoprotein) and collagen fibrils, respectively, during bone resorption [21,22], suggesting that osteoclasts binding to mineralized bone surface is indispensable for osteoclast function. Mature osteoclasts start to degrade the bone and liberate growth factors trapped within the matrix. These factors further recruit MSCs at the resorption sites to differentiate into osteoblasts and form mineralized matrixes which replace the old bone. Under pathological conditions such as rheumatoid arthritis, osteoporosis, and myeloma, osteoclasts become overactive, and bone resorption surpasses new bone formation [16].

During the resorption phase, mature osteoclasts attach to the targeted matrix surface and generate sealing zones between the bone surface and basal membrane via the formation of actin ring, which is a dense belt-like structure making the plasma membrane into a ruffled border. The ruffled border is an active area to hydrolyze and dissolve collagen and other matrix proteins through the secretion of protons and digestive enzymes such as tartrate-resistant acid phosphatase (TRAP), MMP-9, and cathepsin K (CTSK).

### 2.2 Signaling pathways in osteoclast formation

Osteoclasts are formed by the fusion of osteoclast progenitor cells derived from hemopoietic progenitors. M-CSF and RANKL, presented by osteoblasts and osteocytes [16], are two cytokines that are essential and sufficient for osteoclasts formation. M-CSF is necessary for the differentiation of hematopoietic stem cells into the monocyte/macrophage lineage, further facilitates their proliferation and extends their lifespan, RANKL is crucial for osteoclast differentiation and fusion of mature osteoclasts. During the osteoclast differentiation process, osteoclast precursors first differentiate into TRAP-positive mononucleated osteoclasts, then become mature osteoclasts featured with distinctively giant cytomembrane, multiple nuclei through cell-cell fusion [23] (Fig. 1).

**Fig. 2:**
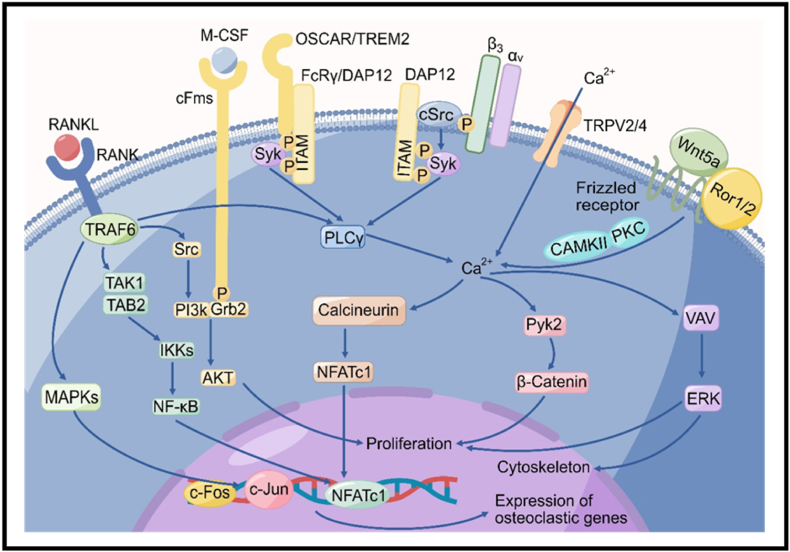
Osteoclast signaling pathways. M-CSF and RANKL signaling are two predominant pathways involved in osteoclast differentiation. The interaction between M-CSF and cFms results in recruitment of PI3K and Grb2, which plays an important role in maintenance of the survival of osteoclast. The RANKL-RANK interaction activates several signaling cascades, including MAPKs, NF-κB, and AKT pathways, to induce osteoclast differentiation, activation and proliferation via the TRAF adaptor proteins (mainly TRAF6). Besides, RANKL signaling can evoke Ca^2+^ oscillations via activation of PLCγ, which induces the release of Ca^2+^ from intracellular Ca^2+^ store sites such as the mitochondria and endoplasmic reticulum, to prompt calcineurin-dependent dephosphorylation and activation of NFATc1, allowing the differentiation of osteoclasts. Ca^2+^ entry through TRP2/4 (Ca^2+^-permeable channels) occurs simultaneously with intracellular Ca^2+^ release, also contributing to the Ca^2+^ oscillations and affecting osteoclast differentiation. Wnt5a can also activate calcium signaling via calcineurin and PKC signals by binding to a receptor complex including Ror 1/2 and a frizzled receptor. As a co-stimulatory way of RANK signaling, Ig-like receptors (OSCAR and TREM2) associate with transmembrane adapter proteins (FcRγ and DAP12), which contain ITA motifs, the phosphorylation of the motifs leads to the activation of PLCγ, Ca^2+^, β-catenin, and ERK signaling, which are critical for osteoclast cell proliferation and cytoskeleton rearrangement. DAP12 can solely work with α_v_β_3_ integrin to regulate the osteoclast cytoskeleton and actin ring formation through activation of PLCγ, VAV and ERK.

To date, one of the most significant breakthroughs in the study of osteoclastogenesis is the finding that osteoprotegerin (OPG), RANKL, and RANK, which belong to the family of biologically related tumor necrosis factor (TNF) receptor (TNFR) proteins, are key cytokines that regulate osteoclasts formation and bone resorption [24]. RANK is a transmembrane signaling receptor expressed on hematopoietic precursor cells. RANKL is secreted by osteoblasts and osteocytes and functions via binding to RANK on the surface of osteoclast precursors (Fig. 2). In addition, the vesicular RANK has been recently reported to reversely bind to osteoblastic RANKL to facilitate bone formation by osteoblasts [25]. OPG is also a soluble protein released by osteoblasts when stimulated by anabolic agents such as bone morphogenic proteins (BMPs) and oestrogens. OPG is considered to prevent osteoclasts formation and osteoclastic bone resorption as a decoy receptor by suppressing RANKL-RANK interaction [16,26].

Except for M-CSF and RANKL, a RANK co-stimulation pathway that includes transmembrane adapter proteins (FcRγ and DAP12), containing intracellular tyrosine-based activation motifs, and Ig-like receptors (OSCAR and TREM2) is also crucial for driving osteoclastogenesis via activating Ca^2+^ signaling [27]. The changes in intracellular Ca^2+^ concentration, termed Ca^2+^ oscillations, play important roles in osteoclast differentiation via activation of nuclear factor of activated T cells c1 (NFATc1) [28]. Simultaneous with Ca^2+^ oscillations, Ca^2+^ entry through transient receptor potential (TRP) channels on plasma membrane is also crucial for osteoclast differentiation [29,30]. In addition to the co-stimulation pathway, DAP12 can associate with α_v_β_3_ integrin to regulate the osteoclast cytoskeleton and actin ring formation [31]. Through binding to a receptor complex including receptor tyrosine kinase-like orphan receptors (Ror 1/2) and a frizzled receptor, Wnt5a can activate Ca^2+^ signaling via calcineurin and PKC signals [32], which is a non-canonical way to affect osteoclast differentiation (Fig. 2).

### 2.3 The role of osteoclasts in bone formation

Following osteoclast-mediated bone resorption, multiple factors that are released from the bone matrix are identified to be beneficial for the establishment of an osteogenic microenvironment through the recruitment of MSCs. Among all the matrix-derived factors associated with the bone resorption, transforming growth factor β (TGF-β) [33,34], vascular endothelial growth factors (VEGF) [35], insulin-like growth factor-1 (IGF-1) [36], and BMP [37] have been extensively studied for their roles in modulating the migration and further osteoblastic differentiation of MSCs. In addition to the above factors derived from bone matrix, osteoclasts can also regulate bone remodeling via direct secretion of biological factors, such as Afamin [38], PDGF-BB [8,39], and chemokine (C-X-C motif) ligand 16 (CXCL16) [40], most of which show a positive ability to affect MSCs recruitment or differentiation of osteoblastic precursor cells. These factors can motivate the migration of MSCs. Other factors such as TRAP, complement component 3a (C3a), hepatocyte growth factor (HGF), cardiotrophin-1 (CT-1), and slit guidance ligand 3 (SLIT3) can promote osteoblastic differentiation and bone formation [41,42]. Conversely, Ephrina2 [43] and Sema4D [7] are inhibiting factors derived from osteoclasts for bone formation (Fig. 1).

## 3 Engineering approaches to manipulate osteoclast behavior

As one of the most important cell types in the process of bone remodeling, the behavior of osteoclasts can be affected by a variety of different stimuli, such as ions, surface properties of biomaterials, and certain drugs. In the following part, we introduce several different strategies that can modulate osteoclast behavior, which may facilitate local manipulation of osteoclast behavior and promotion of bone regeneration.

### 3.1 Manipulate osteoclast behavior by ions released from biomaterials

Recent studies have unveiled the significant impact of various ions on the proliferation and differentiation processes of osteoclasts. Among these, Ca^2+^, a vital biological element in bone remodeling, has garnered extensive attention for its regulatory role in osteoclast behavior. It was found that a rise in physiological levels of extracellular Ca^2+^ significantly reduced the osteoclast activity and bone resorption capacity [44,45]. The activity of osteoclasts is regulated by extracellular Ca^2+^ concentrations through several Ca^2+^-sensing systems such as the voltage-gated Ca^2+^ channels, ryanodine receptors, and Ca^2+^ sensing receptors [46,47], and the affinity of OPN for αvβ3. Osteoclasts are also sensitive to changes in intracellular Ca^2+^. The signaling triggered by RANKL induces Ca^2+^ oscillations, prompting Ca^2+^/calcineurin-dependent dephosphorylation and activation of NFATc1 to influence osteoclastic differentiation in diverse ways [48]. Thus, manipulating intracellular Ca^2+^ levels emerge as a promising approach to influence osteoclast behavior. For instance, employing ATP [49] and intracellular Ca^2+^-elevating compounds such as ionophore A23187 and cyclopiazonic acid [50] stimulated the increase in intracellular Ca^2+^ and inhibited osteoclastic activity.

**Fig. 3:**
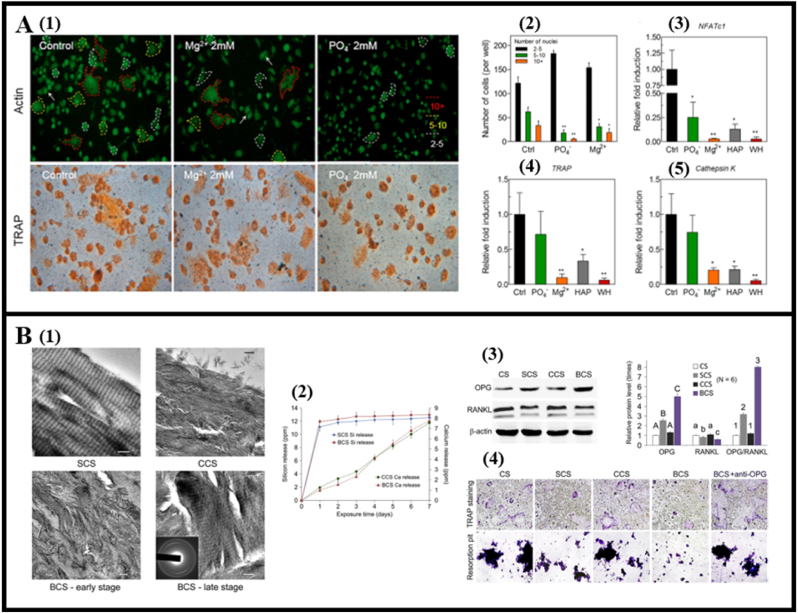
Osteoclast behavior modulated by ions. (A) The effects of Mg^2+^ and PO4^3−^ on osteoclast differentiation of RAW264.7 cells. (Reprinted with permission from Ref. [52], copyright 2017 Elsevier). (A1) Representative images showing Actin-stained cells (top) and TRAP-stained cells (bottom). (A2) The number of multinucleate cells with more than two nuclei. (A3-5) The expression of osteoclast-specific genes NFATc1, TRAP, and CTSK on mRNA level. WH: whitlockite. (B). The effect of silicic acid on osteoclast differentiation of RAW264.7 cells. (Reprinted with permission from Ref. [57], copyright 2015 Elsevier). (B1) TEM images of calcified (CCS), silicified (SCS), and their biphasic (BCS) mineralized collagen scaffolds. (B2) Cumulative release profiles of silicic acid and Ca^2+^ from the collagen scaffolds. (B3) SCS-conditioned MSCs showed up-regulation of OPG expression and down-regulation of RANKL expression; (B4) Effects of scaffold-conditioned MSCs on osteoclastogenesis and osteoclast function of RAW264.7 cells examined by TRAP staining and resorption pit assay.

Another crucial metallic element, magnesium (Mg^2+^), is essential for maintaining bone strength as a micronutrient. Kim and Zhao et al. highlighted the potential of sustained and controlled release of Mg^2+^ to inhibit osteoclast differentiation and promote cancellous bone reconstruction in ovariectomized (OVX) rats [51,52] (Fig. 3A). Furthermore, other metallic elements like gallium (Ga^3+^) [53], strontium (Sr^2+^) [54], manganese (Mn^2+^) [55], and copper (Cu^2+^) have also been observed to modulate osteoclast behavior. These ions influence osteoclast behavior through different mechanisms, such as causing ruffled border dysfunction, leading to osteoclast apoptosis, and regulating intracellular reactive oxygen species (ROS). It is intriguing that all these ions are divalent cations. Zaidi et al. presumed that these ions mimic the inhibitory effect of Ca^2+^ on osteoclasts through an action on a surface membrane “Ca^2+^ receptor” that can also bind other divalent cations [56].

Nonmetallic elements also impact osteoclast behavior. Silicon (Si), an essential trace element in bone regeneration, was found to reduce TNFα-induced NF-κB activation via a microRNA-146a (miRNA-146a) negative feedback loop, thus inhibiting osteoclast differentiation [58]. Kai et al. developed a scaffold containing intrafibrillar silica and apatite, observing upregulated OPG expression and downregulated RANKL expression due to released silicic acid, thereby inhibiting osteoclastic differentiation [57] (Fig. 3B). Phosphate and chloride ions, also nonmetallic elements, effectively modulate osteoclastogenesis when released from biomaterials [59,60]. They were found to restrain osteoclast differentiation by interfering with the electrostatic attraction between RANKL and RANK (Fig. 3A).

In summary, incorporating specific ions into biomaterials offers a promising avenue to restrain osteoclast behavior and enhance bone regeneration. Given their manipulation of diverse signaling pathways, integrating these ions into new bone substitute biomaterials can regulate osteoclast numbers and activity through localized ion release, thereby fostering improved bone regeneration (Table 1).

**Table 1:** Ions released from biomaterials to manipulate osteoclasts.

Ions	Biological Materials	Animal model	Effects on osteoclasts	Reference
**Mg**^**2+**^	GelMA-BP-Mg	–	down-regulate the expression of NFATc1, TRAP and CTSK genes to prevent the number of active osteoclasts	[51]
	whitlockite (WH: Ca_18_Mg _2_(HPO_4_)_2_(PO_4_)_12_)			[52]
**Ga**^**3+**^	Ga-loaded Ca^2+^ phosphates (CaP)	cylindrical defects in rat femora	reduce the number of osteoclasts and the expression level of late osteoclast markers	[53]
**Sr**^**2+**^	strontium-substituted Ca^2+^ phosphate silicate bioactive ceramic (Sr-CPS)	calvarial defects model in OVX rats	inhibit osteoclastogenesis through downregulating NF-κB signal pathway	[54]
**Mn**^**2+**^	Mn-contained β-tricalcium phosphate (Mn-TCP)	femoral defect model of osteoporosis rats	Mn^2+^ released by local implantation of the biomaterial can scavenge ROS via Nrf2 activation and inhibit the formation and function osf osteoclasts	[55]
**Si**	–	–	reduce TNFα-induced activation of NF-κB through miRNA-146a to inhibit osteoclast differentiation.	[58]
			up-regulated the expression of OPG and down-regulated the expression of RANKLvia activation of the p38/MAPK	[57]
**Phosphate**	alcium phosphate cement (CPC)	bilateral rat calvarial defect model	inhibit NF-κB signaling pathway expression via reducing the affinity between RANKL and RANK	[59]

### 3.2 Manipulate osteoclast behavior by biochemical properties of biomaterials

While biomaterials have advanced bone regeneration, their design primarily emphasizes enhancing the osteogenic differentiation of MSCs, leaving limited exploration of the impact of biomaterial properties on osteoclasts. Mamalis et al. discovered that acid-etching implant surface inhibits osteoclast formation and regulates the RANKL-RANK-OPG axis to foster a microenvironment conducive to bone repair [61]. Detsch et al. developed carbonate-containing nanocrystalline Ca^2+^ phosphates, significantly curbing osteoclast differentiation and reducing resorption pits on material surfaces [62]. Surface wettability was also found to promote protein adsorption and deters osteoclastogenesis. Bang et al. observed that a hydrophilic SLA surface inhibited monocyte attachment and suppressed TRAP expression and osteoclastogenesis-related genes [63].

**Fig. 4:**
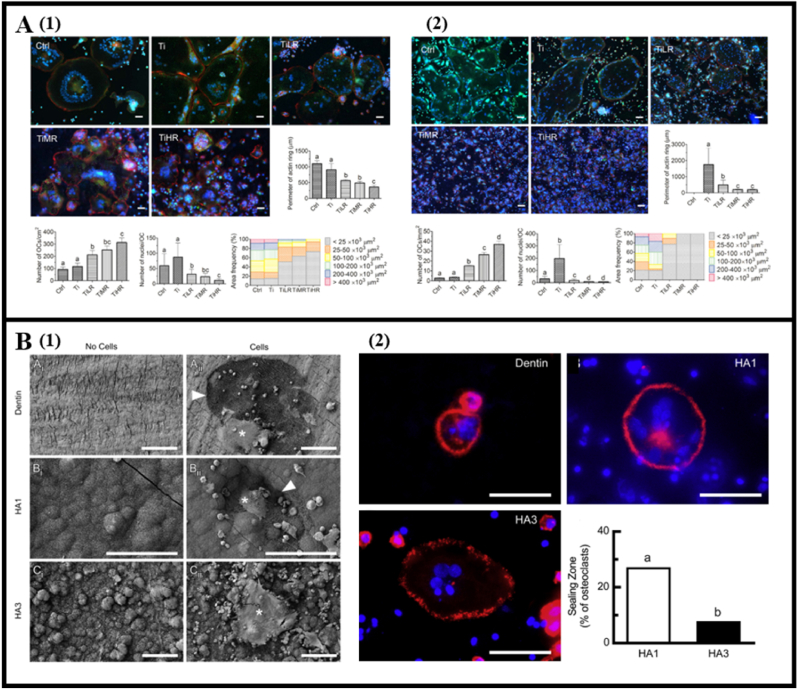
Osteoclast behavior modulated by topographical feature on material surfaces. (A1) RAW264.7 derived osteoclasts and (A2) primary mouse osteoclasts were fewer but exhibited bigger F-actin ring-like structures on smooth surface of titanium disk than those on rougher surfaces of titanium disks with low roughness (TiLR), medium roughness (TiMR), and high roughness (TiHR). (Reprinted with permission from Ref. [64], copyright 2018 The American Chemical Society). (B) Osteoclastic resorption and F-actin organization of rabbit osteoclast precursors after cultured on dentin slices, smoother HAP1, and micro-rough HAP3 surfaces. (Reprinted with permission from Ref. [65], copyright 2013 Elsevier). (B1) Resorption pits indicated by arrowheads and osteoclasts indicated by asterisks. (B2) F-actin (red) staining and DAPI (blue) staining. (For interpretation of the references to color in this figure legend, the reader is referred to the Web version of this article.)

Biomaterial surface roughness also influence osteoclast formation. Zhang et al. noted larger F-actin rings in osteoclasts on smooth surfaces, indicating stronger osteoclast differentiation despite reduced mature osteoclast numbers [64] (Fig. 4A). Similarly, Costa et al. observed heightened osteoclastic activity on smoother surfaces cultured with osteoclast precursors [65] (Fig. 4B). However, Makihira et al. found that roughened titanium promoted osteoclast differentiation via RANK-TRAF6 signaling [66]. Likewise, Brinkmann et al. reported F-actin rings on rough titanium and bone surfaces, contrasting with smooth titanium [67]. These conflicting findings could stem from differences in surface roughness, osteoclast origins, culture methods, and duration. However, it is of note that no matter inhibition or promotion of osteoclast formation on micro-rough surfaces, it is associated with the filamentous actin sealing zones. Given the role of F-actin organization in sealing zones, these studies indicated that focal adhesions which link the cytoskeleton of cells to the biomaterial surface determines osteoclastogenesis.

### 3.3 Manipulate osteoclast behavior by exogenous cells

**Fig. 5:**
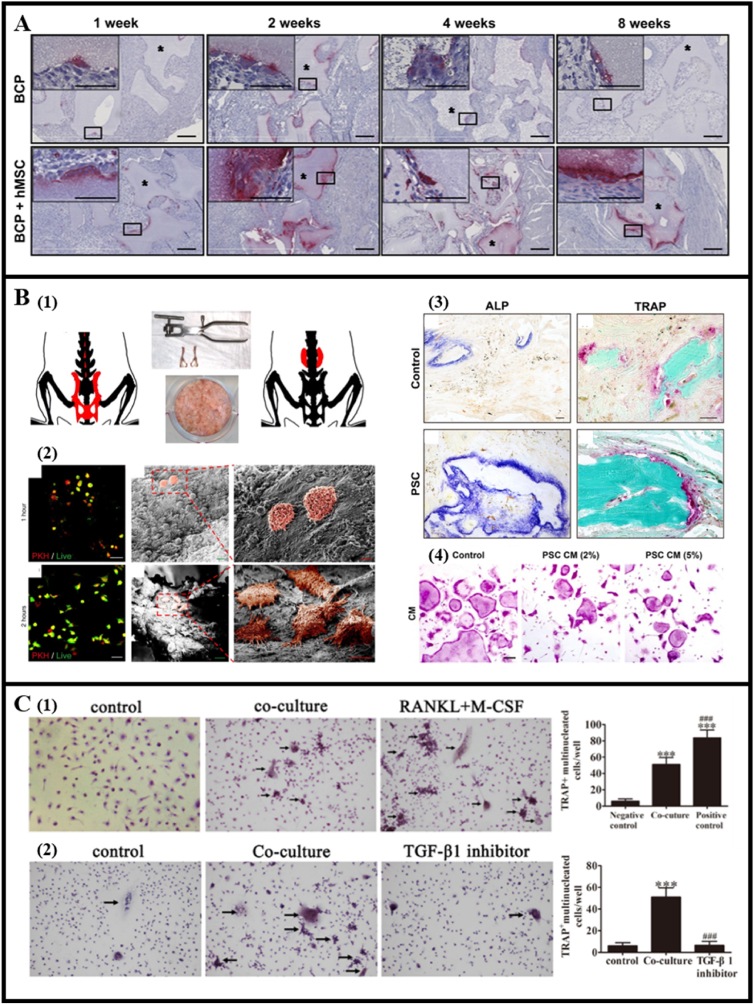
Osteoclast behavior manipulated by exogenous cells. (A) TRAP positive cells increased in BCP implants with hMSCs. (Reprinted with permission from Ref. [70], copyright 2014 Elsevier). (B) PSCs mixed with autograft bone increased osteoblast to osteoclast ratio and promoted bone formation. (Reprinted with permission from Ref. [71], copyright 2020 Oxford University Press). (B1) Graft preparation for posterolateral spine fusion, bone graft harvest area (left), preparation of graft with bone morselizer (middle), and the surgical area (right). (B2) Viability of PKH pre-labeled PSC (appearing red) when seeded on bone graft at 1 and 2 h (left), and the kinetics of PSC adhesion to bone graft (middle and right). (B3) ALP staining showed increased osteoblastic activity while TRAP staining showed no change in osteoclasts formation among spine fusion segments. (B4) Culture of mice BMMs with PSC conditioned medium reduced TRAP-positive cells in vitro. (C) Endothelial cells inhibited osteoclast formation and activity. (Reprinted with permission from Ref. [79], copyright 2018 Karger Publishers). (C1) TRAP staining and the numbers of TRAP-positive multinucleated cells showed that ECs suppressed the differentiation of BMMs into osteoclasts in vitro. (C2) TRAP staining and the numbers of TRAP-positive multinucleated cells showed EC suppressed the differentiation of BMMs into osteoclasts by delivering TGF-β1. (For interpretation of the references to color in this figure legend, the reader is referred to the Web version of this article.)

In addition to the effects of biomaterials on osteoclast behavior mentioned above, exogenous cells, particularly stem cells, modulate osteoclasts through diverse signaling pathways to aid bone repair. Sumi and Lee et al. noted the potential of MSCs to influence osteoclastic differentiation and promote bone regeneration via paracrine factors such as interleukin-6, leukemia inhibitory factor, RANKL, and M-CSF [68,69]. Gamblin et al., observing osteoclastogenesis occurs post-implantation of MSCs and continues even after the death of MSCs, speculated that MSCs induce osteoclast formation and osteoclasts secreted mediators induced subsequent ectopic bone formation [70] (Fig. 5A). However, contradictory findings suggest MSCs aiding bone regeneration by inhibiting rather than promoting osteoclastogenesis [[71], [72], [73], [74]] (Fig. 5B). Cytokines such as OPG, colony-stimulating factor 1, and interleukin-10 and exosomes of MSCs [75] were indicated as main players for the inhibitory effects. Factors such as cell sources, differentiation state, and induction methods likely contribute to the varying effects of MSCs on osteoclasts [76,77]. In tissue development, vascular endothelial (progenitor) cells (ECs) also impact osteoclast behavior in bone regeneration. For instance, ECs were able to modulate the survival, migration, and differentiation potential of osteoclast precursors [78]. The osteoclastogenic effects of ECs were regulated by TGF-β1-mediated Talin-1 expression in macrophages [79]. Mice treated with ECs exhibited increased fracture healing by enhancing recruitment and differentiation of osteoclasts [79]. On the other hand, extracellular vesicles derived from EVs were demonstrated to hinder macrophages from differentiating into osteoclasts by delivering miR-155 and TGF-β1. They disrupt actin ring formation and inhibit the TGF-β1-Talin-1 pathways which resulted in a significant reduction of osteoclasts [79,80] (Fig. 5C).

In summary, while exogenous cells exhibit potential in regulating osteoclast behavior and fostering bone regeneration, additional animal studies are imperative to validate these findings conclusively. Moreover, delineating the precise cell conditions and their potent factors through which exogenous cells modulate osteoclast behavior remains an essential avenue for further investigation.

### 3.4 Manipulate osteoclast behavior by mechanical stimuli

**Fig. 6:**
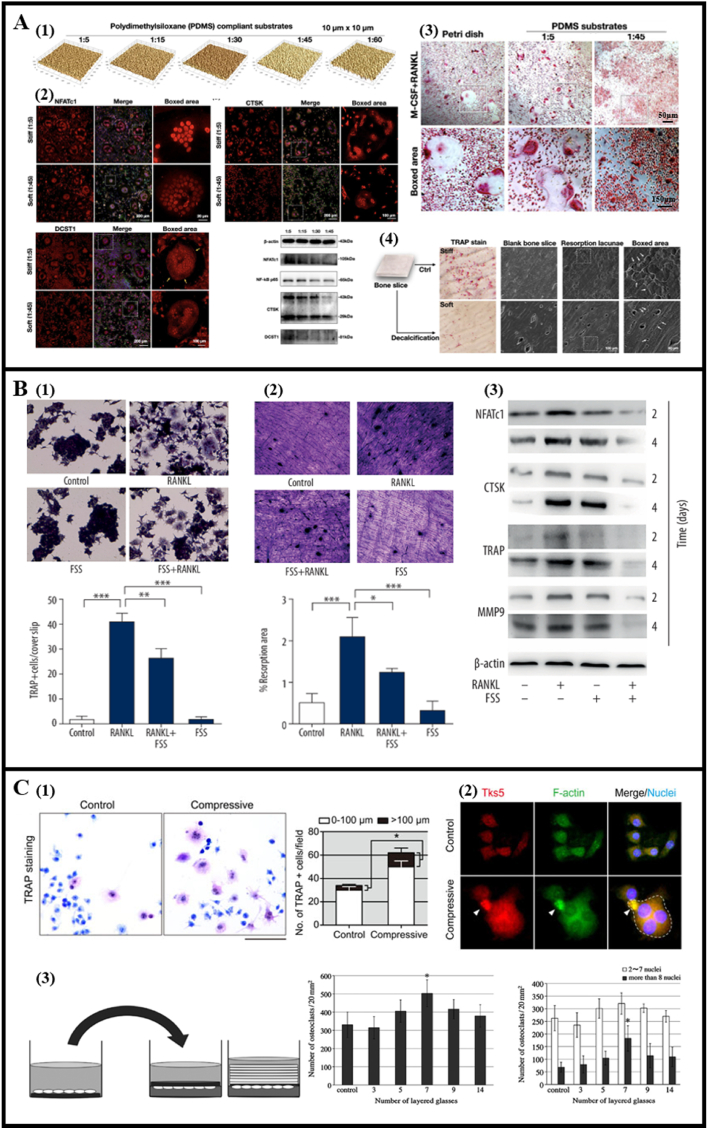
Osteoclast behavior modulated by mechanical stimuli. (A) Effect of material stiffness on osteoclast activity. (Reprinted with permission from Ref. [81], copyright 2021 John Wiley and Sons). (A1) PDMS substrates with different stiffness by changing the mass ratio of the curing agent to the liquid oligomeric base. (A2) Stiffer PDMS substrate upregulated the expression of NFATc1, CTSK, and DCST1 of osteoclasts. (A3) The number of mature TRAP-positive osteoclasts were decreased with the reduction in substrate stiffness (A4) Compared with decalcified bone slices (soft), untreated bone slices (stiff) could significantly promote the formation of TRAP-positive osteoclasts and resorption lacunae. (B) Effect of fluid shear stress (FSS) on osteoclast activity. (Reprinted with permission from Ref. [82], copyright 2020 International Scientific Information). FSS could reverse the formation of RANKL-induced (B1) TRAP-positive cells, (B2) bone resorption area, and (B3) expression of NFATc1, CTSK, TRAP, and MMP9 in protein level of RAW264.7 cells. (C) Effect of compressive force on osteoclast activity. Exposure to compressive force resulted in (C1) more TRAP‐positive cells and (C2) promotion of the expression of Tks5 and F‐actin and the cell fusion (indicated by dash line) of RAW264.7 cells. (Reprinted with permission from Ref. [83], copyright 2018 John Wiley and Sons). (C3) RAW264.7 cells were cultured on slips and reversed them onto the collagen gel layer to receive compressive force, the optimal compressive force to promote osteoclast formation was approximately 300 mg/7 slips. (Reprinted with permission from Ref. [84], copyright 2015 SPANDIDOS PUBLICATIONS).

Bone, as a weight-bearing tissue, undergoes critical regulation through mechanical cues, with bone cells demonstrating mechanosensitivity. Cellular mechanosensors on the membrane and intracellular mechano-signaling proteins significantly influence osteoclast differentiation and function via diverse signaling pathways, converting external mechanical forces into intracellular biochemical cues. The stiffness of the extracellular matrix, a crucial mechanical cue, significantly impacts osteoclast behavior. Wang et al. demonstrated that stiffer substrates (∼4 Mpa) upregulate osteoclast-specific markers and enhance bone resorption capabilities [81] (Fig. 6A). Substrate stiffness regulated integrin αvβ3 and activated downstream intercellular signaling of osteoclasts.

Apart from matrix stiffness, osteoclasts respond to external forces like fluid shear and pressure. Studies exploring the direct impact of fluid shear on osteoclasts indicated conflicting outcomes, where some found it inducing osteoclast formation while others observed inhibition of osteoclast differentiation and marker expression [85,82] (Fig. 6B). Similarly, compression force influences osteoclast behavior. Investigations on collagen gel layers revealed inhibited osteoclast differentiation and fusion, possibly through downregulation of NFATc1 [86]. Contrarily, other studies highlighted the upregulation of factors promoting osteoclast precursor fusion under compressive force [83,84] (Fig. 6C). These experiments shed light on the intricate relationship between osteoclast biology and mechanical stimuli, offering novel pathways to modulate osteoclast behavior. While Ets-1 [83] and mechanosensitive ion channels like Ca^2+^ release-activated Ca^2+^ channel [87], and cation-selective channels [88] are presumed to play a role in osteoclast differentiation responses to mechanical cues, their specific functions warrant further investigation and delineation.

### 3.5 Manipulate osteoclast behavior by drug delivery

The clinical management of bone diseases, often stemming from osteoclast dysregulation, has spurred the development of novel drugs targeting osteoclasts, ranging from natural to synthetic products. However, conventional drug delivery methods for bone healing not only yield undesired side effects but also limit agent bioavailability and concentration due to systemic drug degradation. Thus, combining osteoclast-targeted drugs with tailored local delivery systems emerges as a promising approach for enhancing bone regeneration.

#### 3.5.1 Natural drugs

Natural compounds sourced from plants and animals harbor multiple active components influencing osteoclast behavior (Table 2). Icariin (IC), a flavonoid glycoside from Epimedium, can imped osteoclast differentiation and aided bone regeneration when locally delivered [89,90]. Similar drugs like isoliquiritigenin [91], quercetin [92], baicalin [93] also significantly prevented osteoclast-mediated bone loss and fostered bone formation. The inactivation of RANKL might be the molecular mechanism of these natural drugs. Artemisinin (ARS) [94], dihydroartemisinin (DHA) [95], and artesunate (ART) [96] are another type of natural compounds that can modulate osteoclasts by decreasing the expression of the transcription factors c-Fos and NFATc1, inhibiting the NF-κB signaling pathway, and disrupting the RANKL/OPG balance. Although the effect of these natural drugs in inhibiting osteoclast formation and mitigating osteolysis was proved in different bone disease models, their clinical applications is hampered by the quality control and dose delivery.

**Table 2:** Natural drugs used to manipulate osteoclasts.

Natural drugs	Scaffold materials	Animals	Animal model	Time	Effects on osteoclasts	Reference
**IC**	CPC	female SD rats	bilateral 5-mm diameter calvarium defect in OVX rat model	8 weeks	upregulate the ratio of OPG/RANKL to decrease the differentiation and maturation of osteoclasts	[89]
	IC-loaded HA/alginate porous composite scaffolds	New Zealand rabbits	2.0 cm bone defect in length of the rabbit radius	12 weeks	reduce the number of osteoclasts	[90]
**Isoliquiritigenin**	mesoporous silica nanoparticles	male C57/BL6 J mice	LPS-induced mouse calvarial bone erosion model	7 days	suppress osteoclast-related RANKL signaling pathways and revoke NFATc1 expression	[91]
**Quercetin**	titanium surfaces	female New Zealand White rabbits	rabbit tibia model	8 weeks	inhibit the formation of osteoclasts	[92]
**Baicalin**	poly-caprolactone (PCL)-Poly (lactic-*co*-glycolic acid) (PLGA)-based fibers	SD rat	bilateral 5-mm diameter calvarium defect in rat model	8 weeks	regulate osteoclast differentiation	[93]
**ARS**	–	female mice	OVX mice	12 weeks	inhibit osteoclast formation and function	[94]
**DHA**	–	C57/BL6 J mice	titanium-particle-induced calvarial osteolysis mice model	28 days	suppress AKT/SRC signaling and inhibit osteoclast formation	[95]
**ART**	–	male C57BL/6J mice	iron-overload mice	8 weeks	inhibit osteoclast formation	[96]

#### 3.5.2 Protein and gene-based drugs

Synthetic drugs, known for cost-effectiveness and stability, have integrated with drug delivery systems to modulate osteoclast behavior. BMP-2, a potent osteogenic growth factor, has demonstrated osteoclast modulatory effects when locally delivered with biomaterials such as RhBMP-2, BMP-2 immune complexes (BMP2-ICs), and BMP-2 enhancers in animal models [97]. Similarly, prostate-specific antigen decreased osteoclast number and activity [98].

Except for growth factors and cytokines, genetic tools like short hairpin RNAs (shRNAs) and small interfering RNAs (siRNAs) hold promise for controlling osteoclast-mediated bone resorption. For instance, shRNA targeting Ac45, ATP5B, IGSF23, and Sema3A effectively reduced the number of osteoclasts and bone resorption in infected periapical tissues, arthritis mice, and osteoporotic rats, respectively [[99], [100], [101], [102]] (Table 3). These factors demonstrate effectiveness against specific signaling pathways and have relatively minor side effects, and therefore have more potential to be adopted in future clinical trials.

**Table 3:** Synthetic drugs used to manipulate osteoclasts.

Synthetic drugs	Scaffold materials	Animals	Animal model	Time	Effects on osteoclasts	Reference
**BMP-2**	alginate microbeads	male C57 mice	maxillary right first molar extracted model	2 weeks	BMP2-ICs bind FcγR and activated PLCγ2 phosphorylation to promote osteoclastogenesis	[97]
**IGSF23**	adeno-associated virus (AAV)-shIGSF23	OVX mice	–	3 months	reduce the number of osteoclasts on the bone surface	[100]
**ATP5B (LV-ATP5B) siRNA**	lentivirus particles	male DBA/1 J mice	collagen-induced arthritis	40 days	reduce the differentiation of osteoclast, suppress bone resorption by impaired F-actin formation and decreased levels of adhesion-associated proteins	[101]
**Sema3a plasmid**	plasmid	female mice	OVX	4 weeks	inhibit the differentiation of osteoclast	[102]
**OPG**	polymer microspheres	male SD rats	rodent model of orthodontic tooth movement	28 days	inhibit osteoclasts	[103]
**vanadyl acetylacetonate**	PBS	diabetic BB Wistar rats	femur fracture rat model	6 weeks	reduce osteoclast activity	[104]
**NF-κB decoy ODN**	–	male athymic nude mice	particle-induced bone loss in a murine continuous femoral particle infusion model	28 days	decrease osteoclast activation and reduce osteoclast numbers	[105]
**IPS-02001 (small molecular protein-protein interaction inhibitor)**	–	C57/BL6 male mice	RANKL-induced bone loss in a calvarial model	6 days	disrupt cytoskeleton integrity and inhibit maturation and function of osteoclasts, inhibition of bone resorption via blocking αvβ3 downstream signaling pathways phosphorylation	[106]
		C57/BL6 female mice	OVX	8 weeks		
**CTSK inhibitors (CKIs) L006235**	PLGA nanospheres	Female C57BL6/J mice	mouse unilateral ectopic bone model	3 weeks	suppress osteoclast activity and augment BV	[107]
**Simvastatin**	–	male SD rats	implant surgery in the oral cavity	4 weeks	inhibit the formation of osteoclasts in a concentration dependent manner	[108]
**7ND recombinant protein**	titanium rod	athymic nude male mice	particle-induced osteolysis	4 weeks	inhibit the osteoclast differentiation	[109]

#### 3.5.3 Chemical drugs

**Fig. 7:**
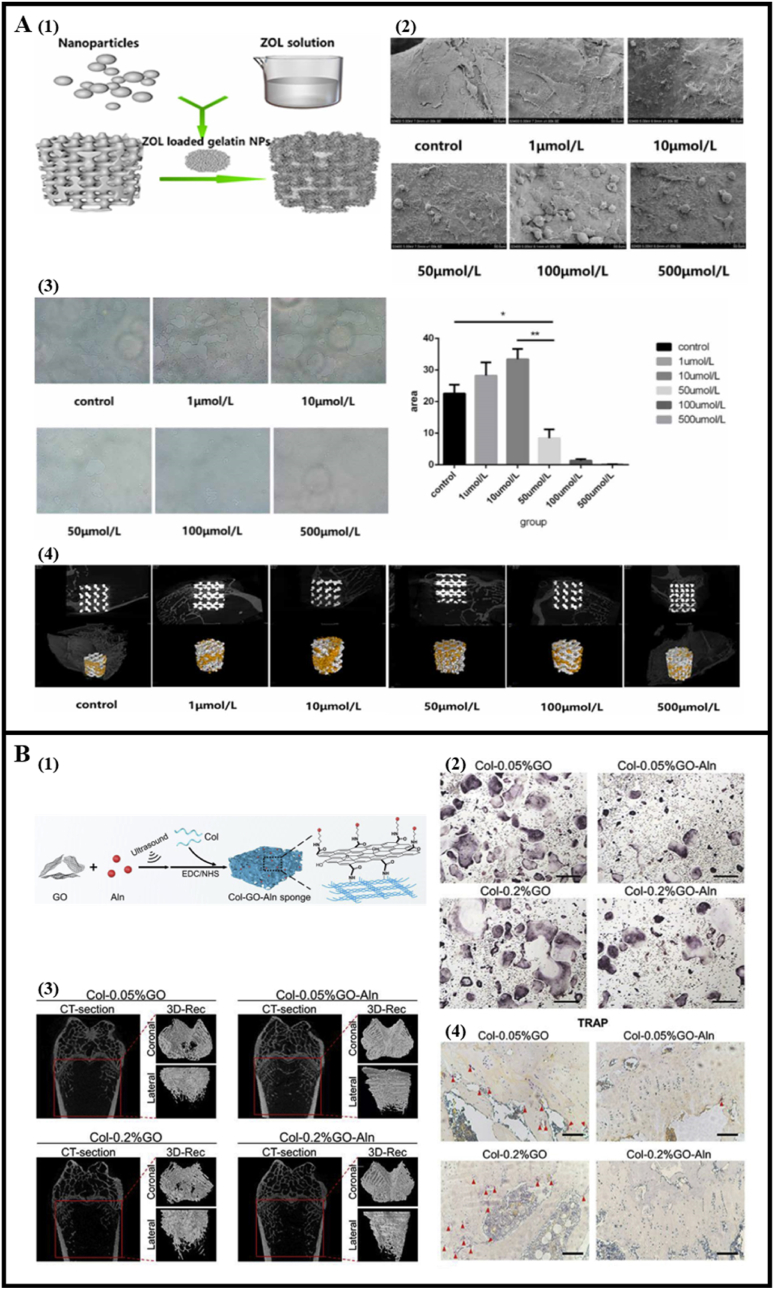
Osteoclast behavior modulated by bisphosphonate. (A) ZOL-loaded scaffolds facilitated bone regeneration through inhibition of osteoclastogenesis. (Reprinted with permission from Ref. [112], copyright 2020 IOP PUBLISHING LTD). (A1) Scheme of the fabrication of ZOL-loaded gelatin NPs integrated porous titanium scaffold. (A2) Morphology of osteoclasts attached on porous titanium scaffolds loaded with different concentrations of ZOL. (A3) The porous titanium scaffolds with high concentration of ZOL (50 μmol/L) inhibited the resorption pits formed by osteoclasts. (A4) micro-CT scans showed high concentration of ZOL-loaded scaffolds induced more bone reconstruction of the femoral condyle defection of OVX rabbits. (B) Col-GO-Aln sponges inhibited osteoclastogenesis; (Reprinted with permission from Ref. [116], copyright 2020 Elsevier). (B1) Scheme of the fabrication procedure and morphology of Col-GO-Aln sponges. Aln released from Col-GO-Aln sponges inhibited osteoclasts formation (B2) in vitro and (B3, B4) in vivo.

Apart from protein and nucleic acid drugs, some synthesized compound molecules can also target specific osteoclast differentiation pathways to intervene in osteoclast behavior. Among them, the most studied are bisphosphonates, whose main principle is to interfere with the mevalonate pathway of osteoclasts to induce apoptosis in osteoclasts. However, this nonselective strategy causes apoptosis of all bone-resorbing cells, thus disrupting necessary bone turnover and leading to atypical femur fractures [[110], [111], [112], [113], [114], [115], [116], [117]] (Fig. 7 & Table 4). In recent years, new synthetic drugs have continued to emerge. Among them, there are those targeting the RANKL/RANK signaling pathway (OPG [103], vanadyl acetylacetonate (VAC) [104]), NF-κB (oligodeoxynucleotide (ODN) [105]), actin cytoskeleton in mature OCs (IPS-02001 [106]), CTSK (L006235 [107], simvastatin [108]), TRAP (recombinant protein 7ND [109]) (Table 3). Although these drugs show a significant inhibitory effect on bone resorption, their specificity, side effects, and the mechanisms promoting bone formation are still unknown. Among all these drugs, targeting OPG may have the most potential, as they can decouple bone resorption from bone formation, thereby inhibiting osteoclasts without affecting bone formation. In addition, a key consideration is how to achieve time and spatial release of these drugs to match the optimal bone regeneration process. Currently used drug delivery systems are mainly focused on conventional materials such as Ca^2+^ phosphate, titanium metals, hydrogels, etc. More advanced types of biomaterials, such as stimuli-responsive hydrogels and nanoparticles, and processing methods such as 3D printing, help further enhance the therapeutic effects of these drugs.

**Table 4:** Bisphosphonates and its analogues used to manipulate osteoclasts.

Drugs	Scaffold material	Animal	Animal model	Time	Effects on osteoclasts	Reference
**Zoledronate (Zol)**	PBS	male SD rats	a rat model of teeth movement under orthodontic force	21 days	inhibit osteoclastogenesis	[110]
	Mg–Nd–Zn–Zr alloy (denoted as JDBM)	SD rats	osteoporotic fracture model	12 weeks	decrease the number of osteoclasts	[111]
	polydopamine-coated porous titanium scaffold	female New Zealand White rabbits	5 mm in diameter defect of OVX rabbits	8 weeks	suppress osteoclastogenesis through proapoptotic function	[112]
	PLGA	male SD rats	bone defect (2 × 4 mm diameter) along the transepicondylar axis	6 weeks	reduce the number of osteoclasts	[113]
	porous titanium/poloxamer 407 hydrogel system	female New Zealand rabbits	cylindrical distal femur bone defect (6.0 mm in diameter and 10.0 mm in depth) of the OVX rabbis	6 weeks and 12 weeks	inhibit the activity of osteoclast	[114]
**Alendronate (Aln)**	PLGA	male Wistar rats	bone defect in the right femur (2.3-mm diameter)	15 days	inhibit RANKL to decrease osteoclasts	[115]
	collagen - graphene oxide (GO) sponges	SD rats	5 mm diameter calvarial defect of OVX rats	3 months	inhibit osteoclastogenesis of monocyte-macrophages	[116]
	polycaprolactone/gelatin (PG)-blended membrane embedded	–	5 mm diameter calvarial defects of OVX rats	4 weeks and 8 weeks	inhibit osteoclast formation	[117]

#### 3.5.4 Exosomes and miRNAs

Intriguingly, cell-derived products, particularly exosomes and miRNAs, have emerged as key regulators of osteoclast behavior. exosomes from human urine stem cells [118] loaded in different nanoparticles and hydrogels have been applied to inhibit osteoclast formation for superior bone formation. These exosomes harbor a diverse range of mRNA, miRNAs, proteins, and lipids, which interfere with the signaling pathways of osteoclast development and consequently inhibit the osteoclast formation and activities. Notably, most of these exosomes are derived from MSCs and exosomes from the adipose tissue MSCs were proved owing the most potential to suppress osteoclast activities due to the high levels of OPG and miR-21‐5p in their exosomes [75]. To advance this strategy utilizing exosomes to modulate osteoclast behavior, one the prospective strategies is to construct genetically-modified cells which highly expressed certain types of proteins and miRNA and subsequently isolate protein or miRNA enriched exosomes. Alternatively, the therapeutic proteins, mRNAs, miRNAs can be directly transfected into exosomes with the aid of electroporation technique and transfection reagents which can significantly elevate the suppressive effects of exosomes on osteoclast formation.

Considering the significant effects of miRNA in osteoclast formation, directly delivering specific miRNA using specific materials to interfere with osteoclasts has great therapeutic potentials. Compared to exosomes containing thousands of miRNAs, this method has higher target specificity. Therefore, in recent years, several miRNAs, including miRNA-124 [119], miRNA-214-3p [120], miRNA-21 [121], miRNA-7b [122], miRNA-31a-5p [123] and miRNA-29a [124], have been used in the treatment of diseases such as osteoporosis, bone defects, and nonunion (Table 5). The delivery materials include liposomes, cationic polymers, gold and silica-based nanoparticles, viral vectors, among which liposomal delivery systems are considered relatively safe and have been used in clinical trials. In these studies, the development of a delivery system specifically targeting osteoclasts also holds important value for treatment. For instance, Wang et al. developed cationic polymers coated with preosteoclasts membranes which can septically target osteoclasts due to the fusion proteins and adhesion molecules from osteoclasts [125]. After intravenously injected, this system substantially reduced the mature osteoclast activities, exhibited higher bone volume (BV), and ameliorated the osteolytic conditions.

**Table 5:** MicroRNAs used to manipulate osteoclasts.

MicroRNAs	Scaffold material	Animal	Animal model	Time	Effects on osteoclasts	Reference
**miRNA-124**	AteloGene Reagent	Lewis rats	adjuvant-induced arthritis in rats	18 days	suppress the differentiation of osteoclast via reducing the expression of human NFATc1	[119]
**miRNA-214-3p**	–	mice	–	2 months	promote osteoclast differentiation	[120]
**miRNA-21**	–	mice	OVX mice	3 months	promote osteoclast function through targeting programmed cell death 4 (PDCD4) and the expression of c-FOS and p-*c*-fos	[121]
**miRNA-7b**	polyethylenimine (PEI) functionalized GO complex	mice	OVX mice	4 weeks	inhibit cell–cell fusion of osteoclast but had no effect on the formation of mononuclear TRAP positive cell	[122]
**miRNA-31a-5p**	exosomes derived from BMSCs	rats	–	3 months	inhibition of miR-31a-5p in exosomes can reduce osteoclastogenesis and bone resorption by elevating RhoA expression at the translational level	[123]
**miRNA-29a**	–	C57BL/6 mice	OVX mice	4 weeks	repress osteoclast formation by regulating PCAF-mediated RANKL and C–X–C motif chemokine ligand 12 (CXCL12)	[124]

## 4 Conclusion and future perspectives

### 4.1 Strategies to decouple bone resorption and bone formation by osteoclasts

In recent studies, there has been a predominant focus on regulating osteoblastic cells to enhance bone regeneration capacity, while only a limited number of studies have explored targeting osteoclasts to modulate the bone remodeling process. Although some reports have attempted to inhibit osteoclast formation for the promotion of bone regeneration, it has been observed in several studies that suppressing osteoclast activities can negatively impact bone formation due to the coupling of osteoclasts and osteoblasts [126,127]. Considering the anabolic effects of osteoclasts on bone formation, even in the absence of bone resorption, there is significant potential in developing novel anti-resorption drugs that can block bone resorption without affecting the anabolic effects of osteoclasts. For instance, the inhibition of CTSK in osteoclasts has shown to reduce bone resorption while enhancing bone formation in an S1P-dependent manner [128]. Consequently, targeting CTSK to stimulate the release of factors from osteoclasts that promote bone formation holds promise as a therapy for addressing bone defects, particularly in compromised patients such as those with osteoporosis or diabetes. This strategy is achieved based on a comprehensive understating of osteoclast developmental biology and their roles in bone remodeling. Osteoclasts arise through cell fusion from mononucleated preosteoclasts to multinucleated mature osteoclasts, in a multistep process mediated by rearrangement of the actin cytoskeleton. Since only mature osteoclasts are mainly responsible for bone resorption through the synthesis of proteases such as CTSK and acids, developing a spatiotemporally selective strategy to inhibit the activity of CTSK and acid-sensing ion channels can separate the anabolic and catabolic effects of osteoclasts without suppressing necessary bone turnover. The other prospective strategy that utilizes osteoclasts for bone formation is to block the maturation of preosteoclasts since mononuclear preosteoclasts have a weak resorption function but can secrete different growth factors to induce angiogenesis and osteogenesis. For instance, transfection of siRNA that enables silencing the expression of dendritic cell‐specific transmembrane protein enables in preosteoclasts caused the blocking of cell-cell fusion and osteoclastogenesis [122]. This impeded bone resorption and augment vascularization and bone formation and therefore can as potential strategies for the treatment of osteoporosis. A similar significant work found that deletion of actin-bundling protein L-plastin (LPL) impeded preosteoclasts fusion by inhibiting filopodia formation but not affected the number of preosteoclasts, which release PDGF-BB to promote bone formation [129,130].

### 4.2 Concerns of using ions to manipulate osteoclasts for bone formation

As we suggested, certain ions offer promising prospects for regulating osteoclast-mediated bone resorption and formation. A notable advantage of ions, as opposed to drugs, is their cost-effectiveness and relative stability during the fabrication process. Furthermore, ion-based therapies generally present fewer safety concerns compared to recombinant proteins or genetic modifications [131,132]. Therefore, the local delivery of these ions through bioactive scaffolds for manipulating osteoclasts holds potential in promoting bone regeneration. Consequently, it is crucial to design scaffolds capable of releasing specific quantities of ions over specific time periods. Various methods, such as ion exchange, solvent casting, salt leaching, electrospinning, and laser sintering, can be utilized to incorporate desired ions into biomaterials. However, exploring the influence of these ions on the metabolism of cell types beyond osteoclasts is crucial. For instance, research indicates that a concentration of 1 mM of Sr ions not only inhibits osteoclast differentiation and formation but also impedes osteoblast activity and reduces cell numbers at the same concentration [133]. Furthermore, the impact of various ions extends to macrophages and endothelial cells, influencing immune response and vascularization processes [134]. These interconnected events collectively contribute to the ultimate outcome of bone formation [135,136]. Consequently, the manipulation of diverse signaling pathways by different ions may pose challenges for in vivo bone regeneration. Thus, further investigation is imperative to deepen our comprehension of how ions regulate biological processes across various cell types, their specific roles in bone formation, and the development of sophisticated bioactive materials capable of delivering specific ions in a spatiotemporally controlled manner.

### 4.3 New engineering approach to manipulate osteoclasts for bone formation

Current engineering approaches to manipulate osteoclast behavior by drugs mainly rely on delivering drugs by bulk materials. This method, however, is limited in clinical application where minimally invasive methods are preferred. Nanoparticles are considered new drug carriers that can target osteoclasts more effectively after injection and allow for intracellular delivery of drugs. However, the low efficiency of drug encapsulation, the burst release of the drug in the initial phase and the limited retention time hampers further practical applications that remains to be solved. In addition, how to specifically target osteoclasts with these drug-loaded nanoparticles is another concern. Song et al. recently introduced endothelial cells derived exosomes into OVX mice and found these exosomes had efficient bone targeting capacity and could inhibit osteoclast activity and alleviate osteoporosis [80]. Diver techniques such as using source-specific exosomes [80], cell membrane coating technology [125], and alendronate conjunction [130] were employed due to their bone targeting capacity. In addition, the local pH changes during bone resorption. Osteoclast formation is stimulated approximately at pH 7.0 and inhibited above pH 7.4 [137]. On the other hand, alkaline pH is beneficial for osteogenic cells [138]. Despite the difficulty to precisely control the local pH around bone formation area in vivo, one pioneering study demonstrated the potential of regulating the pH using special biomaterials to modulate osteoclast behavior [139]. Moreover, the development of pH-responsive polymeric biomaterials for the delivery of drugs to intervene osteoclast activities can be an alternative solution to directly altering local pH. These novel delivery strategies together are expected to provide us with new engineering approach to manipulate osteoclasts for enhanced bone regeneration in the future.

## CRediT authorship contribution statement

**Xin Cheng:** Writing – review & editing, Writing – original draft, Funding acquisition, Data curation, Conceptualization. **Wenzhi Tian:** Writing – review & editing, Writing – original draft, Methodology, Data curation. **Jianhua Yang:** Writing – review & editing, Writing – original draft, Funding acquisition, Data curation. **Jiamian Wang:** Writing – review & editing, Writing – original draft. **Yang Zhang:** Writing – review & editing, Writing – original draft, Supervision, Funding acquisition, Conceptualization.

## Declaration of competing interest

The authors declare that they have no known competing financial interests or personal relationships that could have appeared to influence the work reported in this paper.

## Data Availability

No data was used for the research described in the article.
